# Prevalence of extended-spectrum β-lactamase and carbapenemase-producing *Escherichia coli* from patients, cattle, and environmental sources in northwest Amhara, Ethiopia: a one health approach

**DOI:** 10.1186/s12866-026-05070-z

**Published:** 2026-04-22

**Authors:** Chalachew Genet, Wendemagegn Enbiale, Anna Rommerskirchen, Rajiha Abubeker, Atsbeha Gebreegziabxier Weldemariam, Geremew Tasew, Tazeb Molla, Addisu Melese, Bayeh Abera, Endalkachew Nibret, Abaineh Munshea

**Affiliations:** 1 Health Biotechnology Division, Institute of Biotechnology, Bahir Dar University, Bahir Dar, Ethiopia; 2 Department of Medical Microbiology, Bahir Dar University, Bahir Dar, Ethiopia; 3 Department of Dermatovenereology, Bahir Dar University, Bahir Dar, Ethiopia; 4 Training Center for Neglected Tropical Disease, Arba Minch University, Bahir Dar, Ethiopia; 5 Institute of Medical Microbiology and Hospital Hygiene, Heinrich-Heine- University Düsseldorf, Bahir Dar, Germany; 6 Hirsch Institute of Tropical Medicine, Arsi University, Arsi, Ethiopia; 7 Ethiopian Public Health Institute, Addis Ababa, Ethiopia; 8 Amhara Public Health Institute, Bahir Dar, Ethiopia; 9 Department of Biology, Bahir Dar University, Bahir Dar, Ethiopia; 10 Sine-Hosaena Center, Bahir Dar University, Bahir Dar, Ethiopia

**Keywords:** Carbapenemase, Cattle, Environment, *Escherichia coli*, Extended-spectrum β-lactamase, One Health, Patient, Ethiopia

## Abstract

**Background:**

*Escherichia coli* (*E. coli*) is a Gram-negative, ubiquitous bacterium found in humans, animals, and the environment. Certain strains, notably extended-spectrum β-lactamase-producing *E. coli* (ESBL-PEC) and carbapenemase-producing *E. coli* (CPEC), cause life-threatening intestinal and extraintestinal infections. While some studies exist in patients, data on the prevalence of ESBL-PEC and CPEC among human patients, cattle, and the environment, using a One Health approach, are limited. We aimed to determine the prevalence of ESBL-PEC and CPEC among patients, cattle, and the environment in northwest Amhara, Ethiopia, using a One Health approach.

**Methods:**

A cross-sectional study was conducted from January to August 2025 among 972 study participants, including human patients (508), animals (158), and environmental samples (306). Consecutive sampling was used to select patients and animals. Purposive sampling was used to select environmental samples. Urine or blood from patients, recto-anal mucosal swab from animals, swab, and wastewater from the environment were collected and processed for *E. coli* isolation using standard microbiological methods. ESBL-PEC and CPEC were identified using a combination disk diffusion test and a modified carbapenem inactivation method, respectively, in accordance with the Clinical Laboratory Standard Institute guidelines. Associations were assessed using Chi-square in SPSS, and antimicrobial resistance (AMR) profiles of *E. coli* isolates using a heatmap in R software.

**Results:**

The prevalence of ESBL-PEC among *E. coli* isolated from patients was 52.4% (33/63, 95% CI: 40.3–64.2), while 33.3% (24/72, 95% CI: 23.5–44.8) among environmental isolates and 6.6% (10/151, 95% CI: 3.6–11.8) among cattle isolates. The prevalence of CPEC among patient isolates was 9.5% (6/63, 95% CI: 4.4–19.3) and 2.8% (2/72, 95% CI: 0.8–9.6) in environmental isolates, while no CPEC was detected among cattle isolates. All ESBL-PEC and CPEC isolates were multidrug resistant (MDR). Among non-β-lactam antibiotics tested, ESBL-PEC and/or CPEC isolates showed the highest co-resistance to ciprofloxacin (81.3%), followed by gentamicin (56%), while the lowest was for amikacin (17.3%). Overall, 90.5% (57/63) of *E. coli* from patients, and 81.9% (59/72) from environmental sources, and 29.1% (44/151) from cattle were MDR.

**Conclusions:**

The prevalence of ESBL-PEC and CPEC was high in patients and the hospital environment, whereas ESBL-PEC was low in cattle. No CPEC was detected in cattle. Widespread MDR *E. coli* presence in patients, the environment, and cattle underscores the need for reinforced infection prevention and control measures in hospitals and AMR surveillance based on a One Health approach.

**Supplementary Information:**

The online version contains supplementary material available at 10.1186/s12866-026-05070-z.

## Introduction

*Escherichia coli* (*E. coli*) is a Gram-negative, ubiquitous, genetically versatile bacterium that forms part of the normal microbiota of humans and animals. However, certain strains of *E. coli* have acquired virulence traits that enable them to cause diseases, and are classified as extraintestinal pathogenic *E. coli* (ExPEC) and intestinal pathogenic *E. coli* (InPEC) [1, 2]. The InPEC causes mainly gastrointestinal infections with varying severity depending upon the implicated pathotypes. Meanwhile, the ExPEC is the predominant cause of urinary tract infections (UTIs) and bloodstream infections in humans, as well as mastitis in animals [2–4]. Within a One Health framework, humans, animals, hospital and veterinary clinic environments act as key reservoirs for ExPEC and InPEC [4, 5]. In 2025, a study revealed that zoonotic ExPEC was responsible for approximately 20% of reported UTIs in Southern California, USA [6]. The impact is more profound in developing countries with high *E. coli* disease burden [7].

Antimicrobial-resistant *E. coli*, including resistance to the last resort carbapenem antibiotics used for treating severe bacterial infections, is among the top three Gram-negative bacteria that cause antimicrobial resistance (AMR) associated mortality worldwide [8]. Due to the increasing threat to human and animal health, the World Health Organization (WHO) identified third-generation cephalosporin and carbapenem-resistant *E. coli* as a critical pathogen [9]. Resistance to extended-spectrum β-lactams and carbapenems in *E. coli* is primarily mediated by the production of extended-spectrum β-lactamase (ESBL) and carbapenemase, respectively. ESBL-producing *E. coli* (ESBL-PEC) and carbapenemase-producing *E. coli* (CPEC) cause more economic losses, morbidity, and mortality compared to their non-ESBL and non-carbapenemase-producing counterparts [10–13], and have been associated with outbreaks in various countries [14, 15].

The emergence and dissemination of ESBL-PEC and CPEC result from intricate interactions involving humans, animals, and the environment [16–18]. In addition to high ESBL-PEC and CPEC prevalence in the hospital environment [18, 19], previous studies have documented the reservoir role of diseased and apparently healthy cattle, along with the surrounding environment [20, 21]. These resistant *E. coli* from animals and environmental sources are transmitted to humans primarily by consumption of raw animal foods, direct contact with animals, and drinking of contaminated water [22, 23]. These might have contributed their share to the high prevalence of ESBL-PEC and CPEC among clinical isolates documented in previous studies [24–26]. Consequently, previous studies and the Quadripartite organizations (the WHO, the World Organization for Animal Health, the Food and Agriculture Organization of the United Nations, and the United Nations Environment Program) recommended a One Health approach to tackle the problem in a certain geographical area [16, 27, 28].

**Fig. 1:**
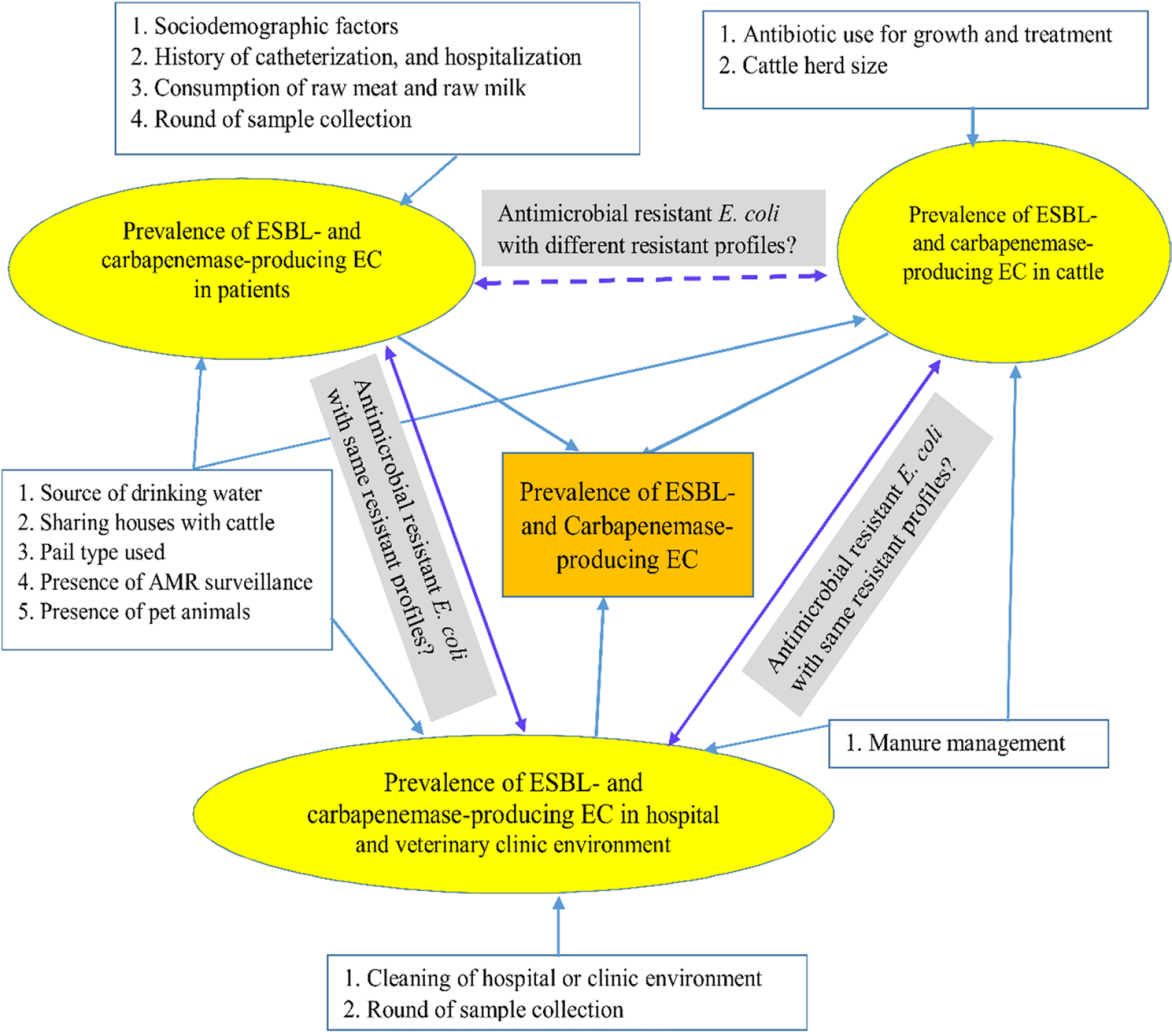
Conceptual framework for prevalence of ESBL- and carbapenemase-producing *E. coli* in northwest Amhara, Ethiopia in 2025: a One Health approach. Key: AMR→ Antimicrobial resistance, ESBL→ Extended-Spectrum β-lactamase, EC→ E. coli

While the role of environment, diseased and apparently healthy animals as a reservoir of antimicrobial-resistant bacteria, including ESBL-PEC and CPEC, is acknowledged [16, 21, 29], there is a scarcity of data in Ethiopia. However, factors contributing to the emergence and spread of ESBL-PEC and CPEC, such as inappropriate antibiotic use, inadequate infection control, unhygienic open-field animal slaughtering, consumption of raw meat, milk, and their derivatives, are widespread in Ethiopia [30–32]. Furthermore, repeated direct contact, sharing houses and sources of drinking water with animals is a common occurrence in Ethiopia, including northwest Amhara [33]. Despite the presence of these favorable grounds, the prevalence of ESBL-PEC and CPEC based on a One Health approach has not been studied in northwest Amhara, Ethiopia. Therefore, we hypothesize that the prevalence of ESBL-PEC in patients and the hospital environment is high but different from the prevalence in animals and the surrounding environment in northwest Amhara, Ethiopia. As indicated in the conceptual framework of the present study (Fig. 1), interactions among patients, cattle, and environment-related factors are hypothesized to influence the prevalence of ESBL-PEC and CPEC in the northwest Amhara, Ethiopia. Therefore, this study, determined the phenotypic prevalence of ESBL- and carbapenemase-producing *E. coli* among patients clinically presumptive for UTI and sepsis, diseased and apparently healthy cattle, and the environment in northwest Amhara, Ethiopia, based on a One Health approach.

## Materials and methods

### Study design, study area and period

A cross-sectional study was conducted in the northwest part of Amhara Regional State, Ethiopia, from January 1 to August 30, 2025. More than half (61%) of households in the Amhara Regional State owned cattle with a mean number of 2.7 (± 1.9) [34]. Moreover, consumption of raw milk and meat is a common practice in Ethiopian households, including the Amhara Regional State [32]. The current study area is home to two public referral hospitals (Felege-Hiwot Comprehensive Specialized Hospital, FHCSH and Tibebe-Ghion Specialized Hospital, TGSH), veterinary clinics, dairy and fattening farms. The FHCSH, with 500 beds, and TGSH, with 493 beds, are among the largest human hospitals in Ethiopia, located in the regional capital city, Bahir Dar. The referral hospitals provide services to residents of the study area, patients referred from different parts of Amhara Regional State and neighboring regions of the country [35].

### Source population

This study included four source populations: Patients with presumptive UTI and patients with presumptive sepsis attending public hospitals (FHCSH and TGSH) in northwest Amhara, Ethiopia; the environment in public hospitals (FHCSH and TGSH) and veterinary clinics; and cattle from farms and public veterinary clinics in northwest Amhara, Ethiopia.

### Inclusion and exclusion criteria

Human study participants who provided written informed consent (or assent for minors) and animals whose owners granted verbal consent were included. Conversely, human study participants or animal study subjects who had received antibiotics within the preceding two weeks were excluded. Moreover, human study participants and cattle owners who did not provide voluntary consent to participate were also excluded.

### Sample size determination and sampling technique

**Fig. 2:**
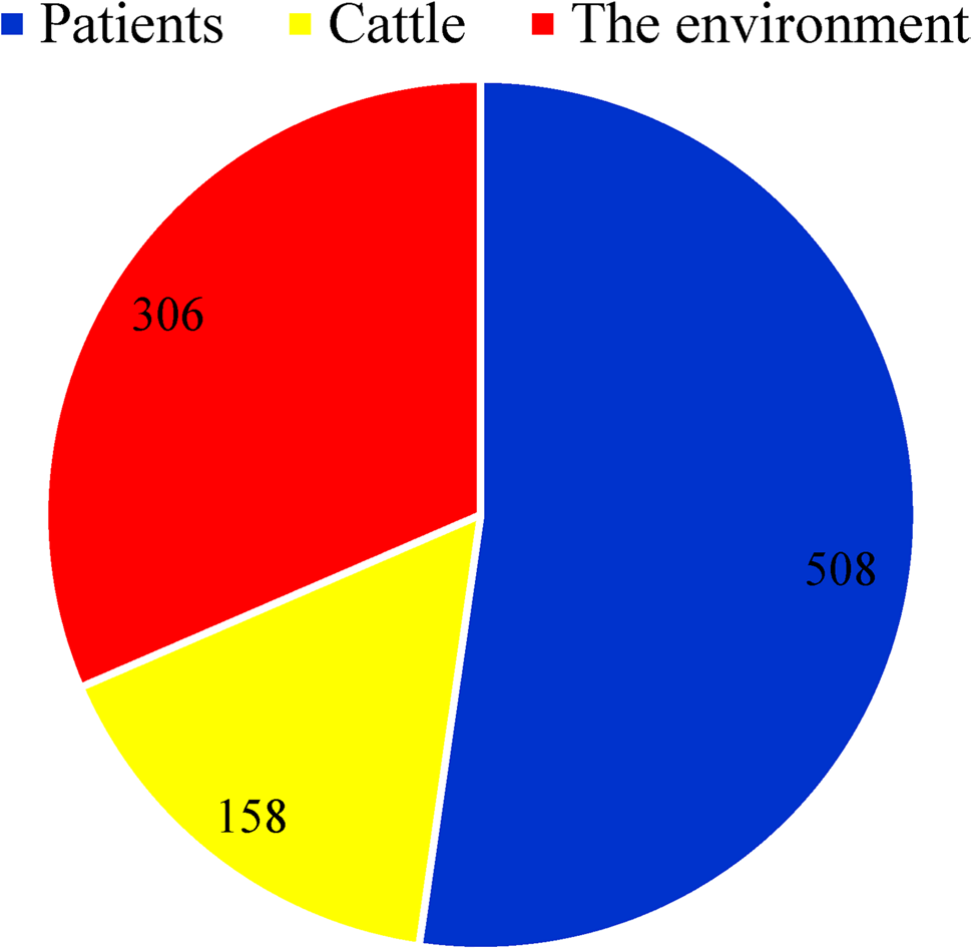
Distribution of the total sample size to patients, animals and the environment

The present study is part of a large research project aimed to determine the prevalence and transmission dynamics of ESBL- and carbapenemase-producing *E. coli* and *K. pneumoniae* among patients, cattle, the environment, and raw cow milk in northwest Ethiopia using a One Health approach. The parent project determined the sample size, collecting 1229 samples using a single population formula. A subsample (*n* = 972) from a larger project was included in this *E. coli* study (Fig. 2).

The consecutive sampling technique was used to include patients and animals in the study, while purposive sampling technique was employed for the environmental sample collection.

### Data collection

Quantitative data for the present study were collected through interviews, and observations using a questionnaire, and checklists, respectively. While clinical (blood and urine) and non-clinical (RAMS, surface swab and wastewater) specimens were collected using appropriate sample collection tools as described below separately for humans, animals and the environment.

### Human sample and data collection

Sociodemographic and related data, urine, and blood were collected from humans. Sociodemographic and related data were collected from patients (sepsis and UTI) and owners of cattle included in this study, using an interviewer-administered structured questionnaire by trained human and veterinary health professionals.

A sterile wide-necked urine cup was used to collect urine samples from patients clinically presumptive for UTI. Ten milliliters (mL) of a clean-catch midstream urine specimen were collected. Similarly, the same amount of urine specimen via a urethral or supra-pubic catheter line was collected from catheterized patients. The collected specimen was delivered to the laboratory at room temperature and processed within 2 h [36]. In the meantime, 2 mL, 5 mL, and 10 mL blood samples were collected from neonates, children, and adults clinically presumptive for sepsis, respectively [37]. Blood and urine samples collected from patients were processed in the microbiology laboratories of FHCSH and TGSH.

### Animal sample and data collection

Data on various aspects of diseased and apparently healthy cattle study subjects, such as sex, presence of a barn, and bedding material, were collected via a checklist. Moreover, the rectoanal mucosal swab (RAMS) sample [38] was collected from one apparently healthy cattle per farm or household from 79 farms, as well as from 79 diseased cattle (diarrheal disease or pneumoniae) attending a public veterinary clinic. For RAMS collection from apparently healthy cattle, a house-to-house survey was conducted in four areas identified as cattle farm zones for milk and meat production in Bahir Dar Special Zone and North Gojjam Zone. To ensure representative sampling, one farm area was selected from each cardinal direction of the study area. After obtaining verbal consent from the farm owner, RAMS were collected from one randomly selected animal per farm. In contrast, diseased cattle attending veterinary clinic were consecutively enrolled for RAMS collection, following verbal consent from their owner. As described in prior studies [38, 39], RAMS samples were collected using sterile cotton swabs moistened with sterile Tryptone Soya Broth (TSB, HiMedia, India). Using a quick in-and-out motion, the moistened swab was inserted 2–5 centimeters (cm) into the anus. After swabbing the entire mucosal surface of the recto-anal junction in a circular motion, the sample was promptly inserted into a test tube containing 3 mL of sterilized TSB.

### Environmental sample and data collection

Data on various aspects of the environment, such as sampling time, and collection rounds, were collected using a checklist by the field teams. The environmental samples, specifically wastewater and surface swab samples, were collected from various FHCSH, TGSH, and veterinary clinic sites (Table 1). The sampling sites were selected considering various factors, including hospital wards serving UTI and sepsis patients, veterinary clinic environments used for providing services to cattle, and accessibility of wastewater manholes. The environmental samples were collected and processed as indicated in prior studies [17, 40]. Briefly, swab samples were collected from veterinary clinic floor surfaces, veterinary clinic tables, hospital bedside tables, inpatient kitchen tables, and serving dishes using a sterile cotton swab moistened in a test tube containing 3 mL TSB. Then, the swab was rotated and rubbed in a 25 cm^2^ area and reinserted into the test tube containing 3 mL TSB. This procedure was repeated three times for each sample to cover the entire sampling area and increase the likelihood of *E. coli* recovery. Prior to each surface swab collection, the 25 cm^2^ sampling template was disinfected with alcohol and allowed to air-dry. In each hospital room, no more than two bedside tables were sampled during each sampling round. For environmental sampling in cattle crush area (a restraint area for cattle) of veterinary clinic, the floor surfaces under the shade were labeled from A to I to ensure representative coverage of the area (supplementary file 1). In each sampling round, one swab sample was collected from each labeled site using the same procedure applied for hospital environmental swab sampling. Similarly, during environmental sampling in the veterinary professionals’ room and the laboratory room of veterinary clinic, four swab samples were collected per sampling round from different tables to obtain representative samples. For hospital wastewater, manholes were selected based on their accessibility, receipt of wastewater from wards managing UTI and sepsis patients, and the inpatient kitchen, as well as their representativeness of the corresponding ward, unit, or kitchen contributing wastewater. Briefly, a 3 mL wastewater sample was collected using a sterile syringe by the grab sampling technique from the wastewater manholes of selected wards, units, the hospital’s main reservoir, and inpatient kitchen wastewater outlets. Then, the sample was immediately transferred into a sterile 50 mL single-use Falcon tube.

The environmental samples and RAMS were transported to the Environmental Health and Biomedical Reference Laboratory of Amhara Public Health Institute, Bahir Dar, Ethiopia, in a cold box for processing. For anticipated delays, the sample was kept at 2–8 °C and processed within a maximum of 6 h.

**Table 1:** Distribution of environmental samples collected from hospitals and veterinary clinic

Organization	Sampling area and type	Samples collected
FHCSH	Bedside table surface swab	70
	Inpatient kitchen table and served dish swab	10
	Wastewater	30
TGSH	Bedside table surface swab	70
	Inpatient kitchen table and served dish swab	10
	Wastewater	40
Veterinary clinic	Cattle crush with shade floor swab	44
	Laboratory room table surface swab	16
	Drug preparation and staff room table surface swab	16
Total		306

FHCSH→ Felege-Hiwot Comprehensive Specialized Hospital, TGSH→ Tibebe-Ghion Specialized Hospital

### Isolation and identification of *E. coli*

*E. coli* was isolated from human samples (urine and blood), animal samples (RAMS), and environmental samples (swab and wastewater) as described below.

### Isolation and identification of *E. coli* from urine samples

A previous protocol was used for this specific study [41], in which a urine sample was mixed by inverting the container, and a 10 µL sample was inoculated into a blood agar plate (BAP, HiMedia, Mumbai, India) within two hours of collection using a calibrated wire loop and incubated at 37 ˚C for 24 h. Plates containing 10^5^ or more colony-forming units (CFU)/mL of urine for non-catheterized and 10^3^ or more CFU/mL for catheterized urine were considered as significant bacteriuria and subcultured further into MacConkey agar plate (MAP, HiMedia, India) to isolate *E. coli.* Urine cultures, which showed < 10^5^ CFU/mL and < 10^3^ CFU/mL for non-catheterized and catheterized urine samples, respectively, as well as mixed urogenital flora growth (growth of more than 2 isolates), were documented as a negative culture result.

### Isolation and identification of *E. coli* from blood samples

The collected blood samples from adults, children, and neonates were directly inoculated into aerobic blood culture bottles with TSB, as it was done in previous studies [23, 37]. The inoculated media were incubated at 37 °C for 24 h. Bottles showing visible growth were subcultured into MAP at 37 °C for 24 h. However, bottles with no visible growth were further incubated up to seven days with daily culture reading before the sample was considered negative.

### Isolation and identification of *E. coli* from RAMS, wastewater and environmental swab samples

As used in a previous study [42], the collected wastewater and RAMS samples were mixed using a vortex for 20 s. Then, 10 µL of the sample was directly inoculated on MAP and incubated at 37 °C for 24 h. Swab samples collected from the hospital and veterinary clinic environments were enriched by incubating at 37 °C for 4 h. Then, 10 µL of the enriched sample was inoculated on MAP and incubated at 37 °C for 24 h. Smooth circular pink colonies suggestive of *E. coli* from clinical, swab, and wastewater samples grown on MAP were subcultured into a BAP and incubated at 37 °C for 24 h. Then, the colony grown on BAP was further identified using a panel of biochemical tests such as sugar fermentation, citrate utilization, motility, urease, hydrogen sulfide production, indole production, and gas production following standard microbiological methods described in the World Health Organization (WHO) guideline [36] and the district laboratory manual in tropical countries [43].

### Antimicrobial susceptibility testing of *E. coli* isolates

The modified Kirby-Bauer disc diffusion method was used to perform antimicrobial susceptibility testing in line with Clinical Laboratory Standard Institute (CLSI) M100-33 edition guideline (M100-Ed33) [44]. Based on prior studies [35, 37] and CLSI guidelines [44], a total of 14 antibiotics from 10 antibiotic categories [45] were selected. These were ampicillin (AMP, 10 µg), amoxycillin-clavulanic acid (AMC, 30 µg), cefazolin (KZ, 30 µg), cefuroxime (CXM, 30 µg), cefotaxime (CTX, 30 µg), ceftazidime (CAZ, 30 µg), imipenem (IPM, 10 µg), meropenem (MRP, 10 µg), gentamicin (GM, 10 µg), Amikacin (AK, 30 µg), ciprofloxacin (CIP, 5 µg), chloramphenicol (C, 30 µg), tetracycline (TE, 30 µg), and Co-Trimoxazole (COT, 25 µg). All antibiotics (except for the last two, which were obtained from HiMedia, India) were obtained from Oxoid (Oxoid, Hampshire, United Kingdom). *E. coli* resistant to at least three antibiotics in different antibiotic categories was considered multidrug resistant (MDR) [45].

### Detection of ESBL-producing *E. coli*

Screening and confirmation of ESBL production were performed phenotypically based on CLSI M100-Ed33 guidelines [44]. Isolates of *E. coli* that showed an inhibition zone of ≤ 22 mm for ceftazidime and/or ≤ 27 mm for cefotaxime were considered as ESBL suspects and further confirmed by the combination disc diffusion test. In this test, isolates suspected of being ESBL producers were tested against ceftazidime (30 µg), ceftazidime-clavulanic acid (30/10 µg), cefotaxime (30 µg), and cefotaxime-clavulanic acid (30/10 µg) antibiotic discs on Muller-Hinton Agar (MHA, HiMedia, India) using the Kirby-Bauer disc diffusion method. After incubation at 37 °C for 18 h, an increase in inhibition zone diameter of ≥ 5 for either combination antibiotic discs with clavulanic acid, when compared with antibiotics tested alone, was identified as an ESBL producer. In addition, ESBL-producing *E. coli* isolates were tested against five antibiotics, considered as possible alternatives to carbapenem for the treatment of ESBL-PEC infections [46, 47], using disc diffusion methods [44]. Antibiotics tested include cefepime (FEP, 30 µg), aztreonam (ATM, 30 µg), cefoxitin (FOX, 30 µg), cefotetan (CTT, 30 µg), and piperacillin-tazobactam (TZP, 110 µg), obtained from Oxoid, UK.

### Detection of carbapenemase-producing *E. coli*

The modified carbapenem inactivation method (mCIM) was used to identify carbapenemase-producing *E. coli* in accordance with the CLSI M100-Ed33 guideline [44]. The Kirby-Bauer disk diffusion method was employed to screen for carbapenemase production with meropenem (10 µg) and imipenem (10 µg) antibiotic discs on MHA. The isolates were regarded as potential producers of carbapenemase if the inhibition zone for meropenem and/or imipenem was ≤ 19 mm and were further confirmed using mCIM. In this method, 1 µL loopful of suspected isolate colony from an overnight incubated BAP was transferred into 2 mL TSB (HiMedia, India) and then vortexed for 10 s. After adding a meropenem (10 µg) antibiotic disc, it was incubated for 4 h at 37 °C. A 0.5 McFarland suspension of meropenem-susceptible *E. coli* American Type Culture Collection (ATCC) 25,922 was evenly swabbed on MHA, and then the meropenem in the TSB was dispensed, followed by incubation for 24 h at 37 °C. The inhibition zone diameter of 6–15 mm or the presence of pinpoint colonies within a 16–18 mm inhibition zone indicated a carbapenemase producer.

### Quality assurance

Various quality assurance procedures were implemented to ensure the quality of the data. Before data collection, data collectors received training. The completeness of the questionnaire was verified both during and after data collection. Regular supervision was also employed to ensure a uniform sample collection process. The sterility test was performed by incubating 5% of uninoculated bacterial culture media from each batch [43]. The *K. pneumoniae* ATCC 700,603 and *E. coli* ATCC 25,922 were used as positive and negative controls for ESBL detection, respectively. Additionally, *E. coli* ATCC 25,922 and *K. pneumoniae* ATCC BAA-1705 were used as negative and positive controls for carbapenemase production, respectively [44]. All reference strains were obtained from the Ethiopian Public Health Institute, Addis Ababa, Ethiopia.

### Data analysis

All data were coded, entered, and analyzed using SPSS version 25 (IBM Corp., Armonk, NY, USA). Based on the complete-case analysis strategy, variables having complete data were considered for analysis. Key variables were summarized using descriptive statistics. Chi-square and Fisher’s exact test were used to see the presence of any association between dependent and independent variables. To control family-wise error rate and minimize false-positive results (Type I error), p-values from these tests were adjusted using a Holm-Bonferroni method in R software version 4.5.1 (R Core Team, 2025). Unadjusted *p*-values < 0.05 were deemed statistically significant when a single comparison was performed. When multiple predictors were tested, Holm-Bonferroni adjusted *p*-values < 0.05 were considered statistically significant. R software version 4.5.1, using the “ComplexHeatmap package”, was also applied to visualize the antimicrobial susceptibility profiles of MDR *E. coli* for 14 tested antibiotics.

## Results

### Prevalence of *E. coli* among patients, cattle and the environment

The overall prevalence of *E. coli* in patients was 12.4% (63/508, 95% CI: 9.9–15.6). While the prevalence in cattle and the environmental samples were 95.6% (151/158, 95% CI: 91.1–97.8) and 23.5% (72/306, 95% CI: 19.1–28.6), respectively. Compared with sepsis patients (6.5%), a significantly higher prevalence was documented in UTI patients (18.7%) (*p* = 0.009 after Holm-Bonferroni adjustment). Although not statistically significant (*p* = 0.145 after Holm-Bonferroni adjustment), the TGSH environment (19.2%) had a higher level of contamination with *E. coli* than the FHCSH environment (9.1%). The environmental sample collected from the inpatient kitchens of both hospitals showed no *E. coli* growth (Table 2).

**Table 2:** Prevalence of E. coli among subgroups in patients, cattle, and the environment

Sample source and study characteristics	Culture result: n (%)		95% CI for prevalence	Chi-square*p*-value^♣ ^	Holm-Bonferroni adjusted p-value
	Negative	Positive^⸙^			
Patient type					
Sepsis (n = 262) UTI (n = 246)	245 (93.5) 200 (81.3)	17 (6.5) 46 (18.7)	4.1-10.2 14.3-24	< 0.001	0.009*
Hospital attended by the patients					
TGSH (n = 254) FHCSH (n = 254)	220 (86.6) 225 (88.6)	34 (13.4) 29 (11.4)	9.7-18.1 8.1-15.9	0.5	1
Cattle health status					
Apparently healthy (n = 79) Diseased (n = 79)	1 (1.3) 6 (7.6)	78 (98.7) 73 (92.4)	93.2-99.8 84.4-96.5	0.053	0.212
Purpose of cattle					
Dairy cattle (n = 85) Beef cattle (n = 58) Farming and beef cattle (n = 15)	4 (4.7) 2 (3.4) 1 (6.7)	81 (95.3) 56 (96.6) 14 (93.3)	88.5-98.2 88.3-99.1 70.2-98.8	0.850	1
Environmental sample source					
Hospital environment (n = 230) Veterinary clinic environment (n = 76)	197 (85.6) 37 (48.7)	33 (14.4) 39 (51.3)	10.4-19.5 40.3-62.2	< 0.001	0.009*
Hospital environment					
FHCSH (n = 110) TGSH (n = 120)	100 (90.9) 97 (80.8)	10 (9.1) 23(19.2)	5-15.9 13.1-27.1	0.029	0.145
Environmental sampling site in hospitals					
Bedside tables (n = 140)	131 (93.6)	9 (6.4)	3.4-11.8	< 0.001	0.009*
Wastewater (n = 70)	46 (65.7)	24 (34.3)	24.3-46		
Inpatient kitchen table and dish (n = 20)	20 (100)	0 (0)	0-13.9		
Environmental sample collection rounds					
Round-1 (n = 77) Round-2 (n = 88) Round-3 (n = 81) Round-4 (n = 60)	56 (72.7) 67 (76.1) 63 (77.8) 48 (80)	21 (27.3) 21 (23.9) 18 (22.2) 12 (20)	18.6-38.1 16.2-33.7 14.5-32.4 11.8-31.8	0.778	1

*Note: → significantly associated, ⸙→ the prevalence was determined by the total number of samples processed for each source, ♣→ Chi-square tests were conducted utilizing observed counts to compare bacterial prevalence among different sample sources, FHCSH→ Felege-Hiwot Comprehensive Specialized Hospital, TGSH→ Tibebe-Ghion Specialized Hospital, UTI→ Urinary Tract Infection

### Prevalence of ESBL-PEC and CPEC among patients, cattle and the environment

**Fig. 3:**
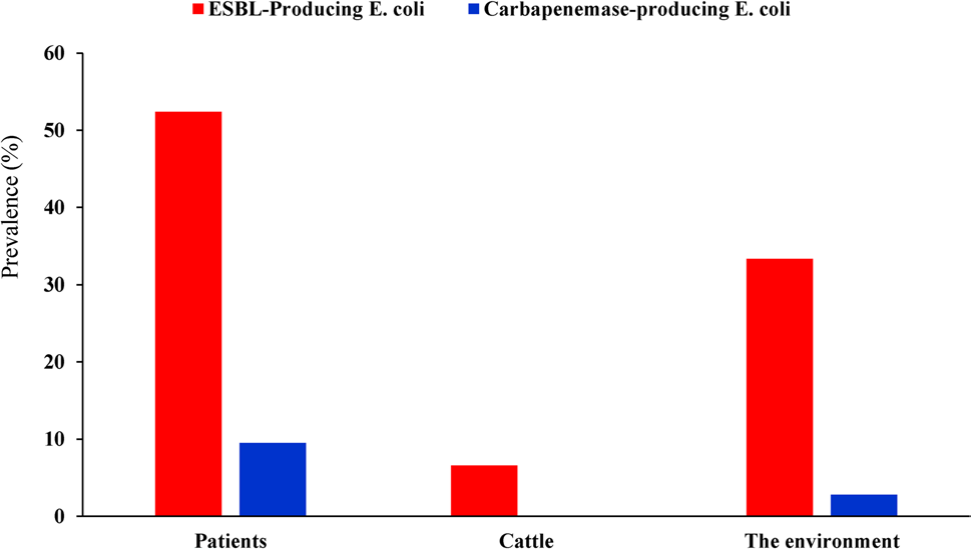
Prevalence of ESBL-PEC and CPEC among *E. coli* isolated from patients, cattle, and the environment

Among the 286 *E. coli* isolates recovered from patients, cattle, and the environmental samples, 23.4% (67/286, 95% CI: 18.9–28.7) were ESBL producers, whereas 2.8% (8/286, 95% CI: 1.4–5.4) were carbapenemase producers. The prevalence of ESBL-PEC showed significant variation among *E. coli* recovered from patients, cattle, and the environment (Chi-square *p* < 0.001), where the prevalence of ESBL-PEC among *E. coli* isolated from patients (52.4%, 95% CI: 40.3–64.2) was approximately eight times higher than the prevalence among *E. coli* recovered from cattle (6.6%). Five of the six (83.3%) carbapenemase-producing clinical isolates were detected in patients attending TGSH. Both CPEC environmental isolates were from TGSH, isolated from bedside tables and wastewater, each accounting for one isolate (Fig. 3).

Our study revealed that 5.1% (4/78, 95% CI: 2.0-12.5) of apparently healthy cattle reared for milk or meat in farms and 8.2% (6/73, 95% CI: 3.8–16.8) of diseased cattle attending veterinary clinic harbored ESBL-producing *E. coli* in their gastrointestinal tract. The prevalence of ESBL-PEC among environmental isolates did not show significant variation in different rounds of sample collection (adjusted *p* = 1). Though the prevalence of ESBL-PEC did not have a significant difference between FHCSH and TGSH environmental isolates (adjusted *p* = 1), the prevalence among *E. coli* isolates in the environment of hospitals (63.6%) was significantly higher than the prevalence in veterinary clinic environment (7.7%, adjusted *p* = 0.008). The present study showed that two-thirds of *E. coli* (66.7%) recovered from hospital bedside table surfaces were ESBL producers. (Table 3).

**Table 3:** Prevalence of ESBL-PEC among *E. coli* isolated from different subgroups of patients, cattle, and the environment

Variables	ESBL prevalence: *n* (%)	95% CI for prevalence	Chi-Square *p*-value	Holm-Bonferroni adjusted *p*-value
Patients				
Sepsis (*n* = 17) UTI (*n* = 46)	9 (52.9) 24 (52.2)	31-73.8 38.1–65.9	0.957	1
Patients				
Attending TGSH (*n* = 34) Attending FHCSH (*n* = 29)	15 (44.2) 18 (62.1)	28.9–60.6 44.0-77.3	0.155	0.93
Cattle health status				
Apparently healthy (*n* = 78) Diseased (*n* = 73)	4 (5.1) 6 (8.2)	2.0-12.5 3.8–16.8	0.445	1
Purpose of cattle				
Dairy cattle (*n* = 81) Beef cattle (*n* = 56) Dairy, farming & beef cattle (*n* = 14)	5 (6.2) 5 (8.9) 0 (0)	2.7–13.7 3.9–19.3 0-19.3	0.472	1
Environmental sample source				
Hospital environment (*n* = 33) Veterinary clinic environment (*n* = 39)	21 (63.6) 3 (7.7)	46.6–77.8 2.7-2.0	< 0.001*	0.008
Environmental sample source				
Bedside table (*n* = 9) Wastewater (*n* = 24) Veterinary clinic (*n* = 39)	6 (66.7) 15 (62.5) 3 (7.7)	35.4–87.9 42.7–78.8 2.7–20.3	< 0.001*	0.008
Hospital environment				
FHCSH (*n* = 10) TGSH (*n* = 23)	7 (70) 14 (60.9)	39.7–89.2 40.8–77.8	0.616	1
Environmental sample collection rounds				
Round-1 (*n* = 21) Round-2 (*n* = 21) Round-3 (*n* = 18) Round-4 (*n* = 12)	7 (33.3) 10 (47.6) 5 (27.8) 2 (16.7)	17.2–54.6 28.3–67.6 12.5–50.9 4.7–44.8	0.298	1

### Antibiotic susceptibility profiles of ESBL-PEC and CPEC against carbapenem-sparing antibiotics

**Fig. 4:**
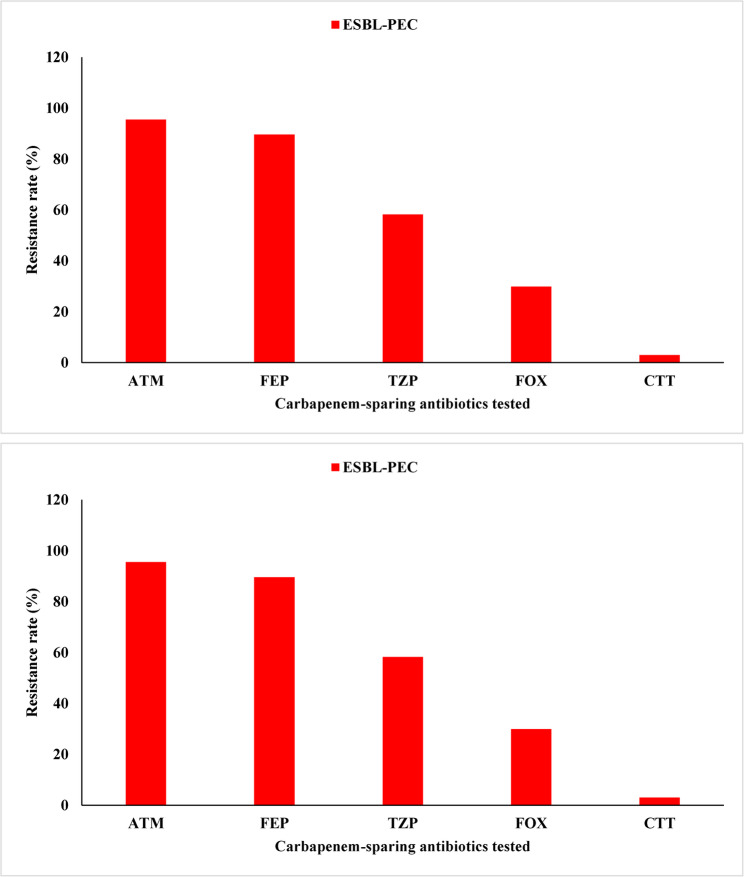
The resistance profiles of ESBL-producer *E. coli* isolates against five carbapenem-sparing antibiotics. Note: ATM: Aztreonam, CTT: Cefotetan, ESBL-PEC: Extended-Spectrum β-lactamase Producing E. coli, FEP: Cefepime, FOX: Cefoxitin, TZP: Piperacillin

ESBL-producing *E. coli* isolates were tested against five carbapenem-sparing antibiotics. The vast majority of ESBL-PEC isolates were resistant to cefepime (89.6%, 60/67) and aztreonam (95.5%, 64/67). Meanwhile, 97% (65/67) of ESBL-producing *E. coli* were susceptible to cefotetan (Fig. 4).

**Fig. 5:**
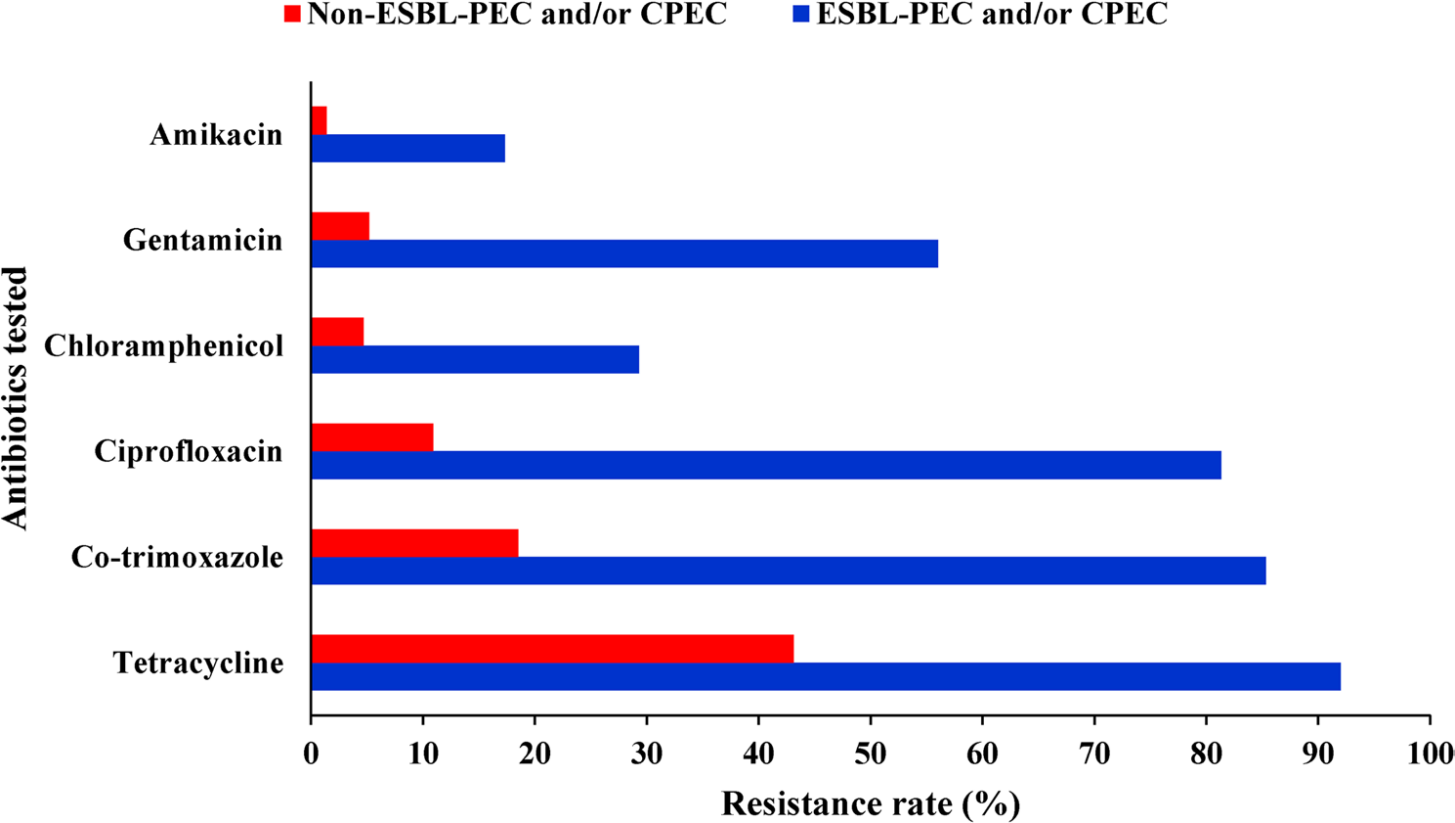
The co-resistance of ESBL-PEC and/or CPEC versus non-ESBL-PEC and/or CPEC to non-β-lactam antibiotics

The resistance levels of ESBL- and/or carbapenemase-producer and non-producer *E. coli* isolates were assessed against six non-β-lactam antibiotics. *E. coli* that were ESBL- and/or carbapenemase producers revealed a higher resistance rate than non-producers to gentamicin (56% versus 5.2%) and ciprofloxacin (81.3% versus 10.9%), respectively. Conversely, the lowest resistance was observed for amikacin, with rates of 17.3% and 1.4% among ESBL- and/or carbapenemase-producing and non-ESBL- and/or carbapenemase producing *E. coli* isolates, respectively (Fig. 5).

### Antibiotic susceptibility profiles of *E. coli* isolated from patients, the environment and cattle

In the present study, 286 *E. coli* isolates were tested against 14 antibiotics from 10 antibiotic categories. The highest resistance rate of *E. coli* isolated from patients, the environment, and cattle was for cefazolin, with 100%, 86.1%, and 81.5%, respectively. Meanwhile, 90.5%, 97.2%, and 99.3% of *E. coli* isolated from patients, the environment, and cattle were susceptible to meropenem, respectively (Table 4).

**Table 4:** Antimicrobial susceptibility profile of *E. coli* isolated from patients, the environment, and cattle

Antibiotic category	Antibiotics tested	Resistant *E*. *coli*: *n* (%)			Total resistant E. coli: *n* (%)
		Patient isolate: *n* (%)	Environmental isolate: *n* (%)	Cattle isolate: *n* (%)	
Penicillins	Ampicillin	59 (93.7)	60 (83.3)	67 (44.4)	186 (65)
Penicillin-β-lactamase inhibitor	Amoxicillin-clavulanic acid	53 (84.1)	48 (66.7)	31 (20.5)	132 (46.2)
Non-extended-spectrum cephalosporins (1st and 2nd generation)	Cefazolin	63 (100)	62 (86.1)	123 (81.5)	248 (86.7)
	Cefuroxime	56 (88.9)	58 (80.6)	96 (63.6)	211 (73.8)
Extended-spectrum cephalosporins (3rd and 4th generation)	Ceftazidime	43 (68.3)	25 (34.7)	7 (4.6)	75 (26.2)
	Cefotaxime	44 (69.8)	31 (43.1)	9 (6)	84 (29.4)
Carbapenems	Meropenem	6 (9.5)	2 (2.8)	1 (0.7)	9 (3.1)
	Imipenem	8 (12.7)	2 (2.8)	0 (0)	10 (3.5)
Aminoglycosides	Gentamicin	35 (55.6)	17 (23.6)	1 (0.7)	53 (18.5)
	Amikacin	5 (8.6)	11 (15.3)	0 (0)	16 (5.6)
Fluoroquinolones	Ciprofloxacin	45 (71.4)	31 (43.1)	8 (5.3)	84 (29.4)
Tetracyclines	Tetracycline	49 (77.8)	59 (81.9)	52 (34.4)	160 (55.9)
Folic acid synthesis inhibitors	Co-trimoxazole	48 (76.2)	35 (48.6)	20 (13.2)	103 (36)
Phenicols	Chloramphenicol	11 (17.5)	14 (19.4)	7 (4.6)	32 (11.2)

### Multidrug resistance profile of *E. coli* isolated from patients, the environment, and cattle

**Fig. 6:**
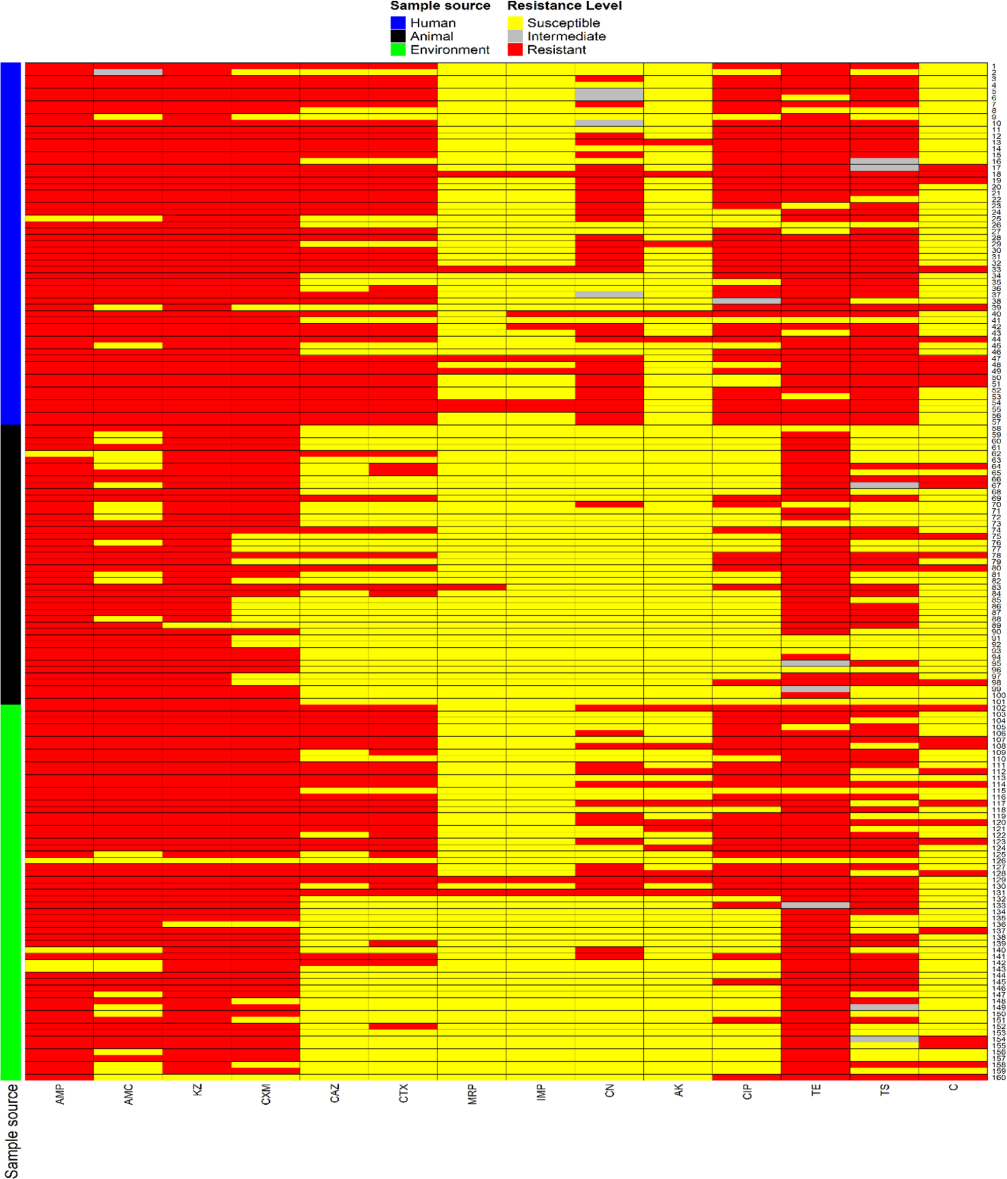
A heatmap showing the distribution of multidrug-resistant *E. coli* isolated from patients, cattle, and the environment. Note: AK→ amikacin, AMC→ amoxycillin-clavulanic acid, AMP→ Ampicillin, C→ chloramphenicol, CAZ→ ceftazidime, CIP→ ciprofloxacin, COT→ Co-Trimoxazole, CTX→ cefotaxime, CXM→ cefuroxime, GM→ gentamicin, IPM→ imipenem, KZ→ cefazolin, MRP→ meropenem, TE→ tetracycline

The MDR rate of *E. coli* was 90.5% (57/63) for patient isolates, 81.9% (59/72) for environmental isolates, and 29.1% (44/151) for cattle isolates. All ESBL-PEC and CPEC and 40.3% (85/211) of non-producer isolates were MDR. As indicated in the heatmap of MDR *E. coli*, 87.7% (45/57) of patients, 45.8% (27/59) of environmental, and 11.4% (5/44) of cattle MDR isolates were resistant to 8 or more antibiotics (Fig. 6).

### Association between different factors with ESBL-producing *E. coli*

The Chi-square test was used to assess the association between patient-, environment- and cattle-related variables and ESBL-producing *E. coli*. Among 13 independent variables, only the environmental sample source showed a significant association with ESBL-PEC colonization (*p* = 0.013 after Holm-Bonferroni adjustment) (Table 5).

**Table 5:** Association between source-related variables and ESBL producing *E. coli* isolates

Variables	Category (*E*. *coli* isolated, *n*)	ESBL result		Chi-Square *p*-value	Holm-Bonferroni adjusted *p*-value
		Producer: *n* (%)	Non-producer: *n* (%)		
Environmental sample from hospitals	FHCSH (*n* = 10) TGSH (*n* = 23)	7 (70) 14 (60.9)	3 (30) 9 (39.1)	0.710^♣^	1
Environmental sample source	Hospital environment (*n* = 33) Veterinary clinic environment (*n* = 39)	21 (63.6) 3 (7.7)	12 (36.4) 36 (92.3)	< 0.001	0.013*
Environmental sample collection round	Round-one (*n* = 21) Round-two (*n* = 21) Round-three (*n* = 18) Round-four (*n* = 12)	7 (33.3) 10 (47.6) 5 (27.8) 2 (16.7)	14 (66.7) 11 (52.4) 13 (72.2) 10 (83.3)	0.298	1
Cattle health status	Diseased (*n* = 73) Apparently health (*n* = 78)	6 (8.2) 4 (5.1)	67 (91.8) 74 (94.9)	0.524^♣^	1
Cattle herd size	Small, < 10 cattle (*n* = 107) Large (> 10 cattle) (*n* = 44)	9 (8.4) 1 (2.3)	98 (91.6) 43 (97.7)	0.282	1
Cattle sharing house with humans	Yes (*n* = 36) No (115)	4 (11.1) 6 (5.2)	32 (88.9) 109 (94.8)	0.251	1
Hospitals attended by the patient	FHCSH (*n* = 29) TGSH (*n* = 34)	18 (62.1) 15 (44.1)	11 (37.9) 19 (55.9)	0.155	1
History of hospitalization in last one year	Yes (*n* = 35) No (*n* = 27)	19 (54.3) 14 (51.9)	16 (45.7) 13 (48.1)	0.849	1
Patient share house with cattle	Yes (*n* = 13) No (*n* = 15)	10 (76.9) 9 (60)	3 (23.1) 6 (40)	1^♣^	1
Raw meat consumption habit by patients	Yes (*n* = 44) No (*n* = 19)	26 (59.1) 7 (36.8)	18 (40.9) 12 (63.2)	0.105	1
Raw milk consumption habit by patients	Yes (*n* = 24) No (*n* = 39)	16 (66.7) 17 (43.6)	8 (33.7) 22 (56.4)	0.075	0.9
Patients have dog and/or cat	Yes (*n* = 46) No (*n* = 13)	25 (67.6) 6 (63.6)	21 (32.4) 7 (36.4)	0.601	1
Milk pail type used by patients	Made of calabash (*n* = 9) Made of plastic or metal (*n* = 27)	3 (33.3) 17 (63)	6 (66.7) 10 (37)	0.146^♣^	1

*→ significantly associated, ♣→ Fisher’s exact test p-value, FHES: Felege-Hiwot Comprehensive Specialized Hospital, TGSH: Tibebe-Ghion Specialized Hospital

## Discussion

*E. coli* is a common inhabitant of various environmental niches and part of the intestinal microbiota in humans and animals [48, 49]. This was substantiated in our study, where one-fourth of the environmental samples (23.5%) and the intestinal tract of almost all cattle (95.6%) harbored *E. coli*. *E. coli* present in the environment and in the intestinal flora of animals can contaminate beef and dairy products due to unhygienic slaughtering and milking practices, poor personal hygiene, and environmental contamination in northwest Amhara, Ethiopia [50]. Consumption of raw or undercooked beef and dairy products, a common practice in Ethiopia [32], further facilitates the transmission of this potentially pathogenic bacterium [51], leading to life-threatening infections, such as sepsis and UTI [52, 53].

The prevalence of *E. coli* in the present study was 18.7% among UTI patients and 6.5% among sepsis patients. The prevalence observed among UTI patients was comparable with findings from our previous study conducted in the same study area in 2019 (15.5%) [37]. However, the prevalence observed among sepsis patients (6.5%) showed a 3-fold increase compared to the prior study, 2.1% [37]. This rise may be partly attributed to the increasing incidence of *E. coli*-associated sepsis [54]. Persistent environmental contamination with *E. coli* in hospital settings across all sampling rounds in our study, coupled with the prolonged non-growth state survival ability of *E. coli* in the environment [55], may also have contributed to the increased prevalence of *E. coli* among sepsis patients.

In our study, the prevalence of ESBL-PEC was 52.4% in patients (52.9% in sepsis patients and 52.2% in UTI patients), 6.6% in cattle, and 33.3% in the environment (63.6% from hospital environment and 7.7% from veterinary clinic environment). The high prevalence of ESBL-PEC in patients and the hospital environment, together with substantial prevalence in animals and their environment, supports our hypothesis and conceptual framework and may suggest ongoing circulation across the human-animal-environment interface in northwest Amhara, Ethiopia. The prevalence of ESBL-PEC in patients and environmental samples in the present study was markedly higher than that reported globally [56, 57], in sub-Saharan countries [16], and in individual country reports from Uganda [58] and Ghana [59]. The higher prevalence in our study may be explained by differences in the environmental sampling strategies, study population characteristics, differences in infection prevention and control measures, and the widespread availability and inappropriate use of antibiotics in Ethiopia [60]. The present study also documented persistent ESBL-PEC contamination in the hospital environment. Patients infected with ESBL-PEC more frequently consume raw meat and milk than those infected with non-ESBL *E. coli*. These factors may facilitate the emergence and spread of extended-spectrum cephalosporin- and carbapenem-resistant *E. coli*, as animals and the environment serve as primary reservoirs [16, 17]. The prevalence of ESBL-PEC among cattle in our study was 6.6%. This was fairly comparable with 3.7% prevalence reported in Somalia, Ethiopia [61], but considerably lower than the pooled global estimates from 2019 (26.3%) [57], and 2023 (33.5%) [56], as well as those reported in Sub-Saharan African countries, namely, Burkina Faso (76.5%) [62], Uganda (55.4%) [58] and Ghana (22.5%) [59]. Although antibiotic use in food animals is increasing in developing countries, including Ethiopia [63–65], the lower prevalence observed in our study may reflect differences in ESBL detection method, variation in study population, and limited access to or use of expanded spectrum cephalosporins in Ethiopian animal farms [65, 66], a driving factor for the development of ESBL-mediated resistance [67].

In the present study, the prevalence of CPEC was 9.5% in patients and 2.8% in the environmental samples. Compared with the 1.8% CPEC prevalence reported from the same area in 2014 [68], our finding indicates a more than fivefold increase. The prevalence of CPEC among patients in this study was also higher than previously reported in Addis Ababa, Ethiopia (0%) [69], Djibouti (2.8%) [70], and Burkina Faso (2%) [71]. Furthermore, the prevalence of CPEC among UTI patients in our study (52.2%) was substantially higher than the 3.42% pooled prevalence reported for Amhara Regional State in 2025systematic review [72]. This marked increase may be attributed to inappropriate use of antibiotics [60, 73, 74] and persistent contamination of the hospital environment observed across all rounds of sample collection in the present study.

Unlike our findings, where no CPEC was detected in cattle, previous studies across Africa have reported varying levels of CPEC in cattle and their surrounding environment [70, 75]. Such disparities may be attributed to differences in farm hygiene, infection prevention and control practices, livestock population, and use of carbapenems in animal farms.

In our study, 89.6% and 58.2% of ESBL-PEC isolates were resistant to cefepime and piperacillin-tazobactam, respectively, both of which are listed as the watch group in the WHO antibiotic list [76] and used as a carbapenem-sparing strategy to treat ESBL-producing bacterial infections [46]. These antibiotics, mainly piperacillin-tazobactam, are designated as the preferred choice in the WHO and Ethiopia essential medicines list for the treatment of severe infections, including complicated intra-abdominal and hospital-acquired infections [77, 78]. Our finding suggests that cefepime and piperacillin-tazobactam may have limited therapeutic utility for ESBL-PEC infections in our study area. Conversely, the majority of isolates remained susceptible to cefotetan (97%). However, cefotetan is not listed on the Ethiopian essential medicines list [77], and its availability and cost may limit its utility in routine clinical practices in Ethiopia. Its potential role warrants evaluation in the context of local formularies and access.

The present study revealed that ESBL-PEC and CPEC isolates exhibited higher rates of co-resistance to non-β-lactam antibiotics compared to non-producer isolates. The co-resistance level of ESBL-PEC to Co-trimoxazole, gentamicin, and amikacin was 85.3%, 56%, and 17.3%, respectively. These were fairly comparable to those reported previously [79]. However, the co-resistance to ciprofloxacin was notably higher in our study (81.3%) compared to the prior study (52.2%) [79]. This could be explained by the carriage of multiple resistant genes on plasmids, conferring resistance to different classes of antibiotics, as documented in a previous study [80]. However, among the tested non-β-lactam antibiotics, amikacin showed the lowest co-resistance rate, suggesting that it may still be considered a potential therapeutic option for ESBL-PEC infection in our study area.

MDR *E. coli* is a growing public health threat in humans, animals, and the environment [81]. The overall MDR rate of *E. coli* in our study was 55.9%. Among *E. coli* isolated from patients and environmental samples, 90.5% and 81.9% were MDR, respectively. Besides, one-third (29.1%) of cattle carried MDR *E. coli* in their gastrointestinal tract. Our findings indicate a high prevalence of MDR *E. coli* among the human-animal-environment interface in northwest Amhara, Ethiopia. The overall MDR rate in our study was nearly three times higher than the global MDR *E. coli* data reported in 2019 [57]. The MDR rate of *E. coli* isolated from patients in our study (90.6%) was higher than the pooled national MDR data (79.17%) [82], MDR data for Amhara Regional State (22.8%) [83], and the MDR data for Mekelle, Ethiopia (80.4%) [84]. Moreover, our finding was higher than prior reports from Ghana, 42.6% [85], and the USA, 18.9% [25]. The MDR rate of *E. coli* isolated from cattle in our study (29.1%) was higher than the prior report from South Africa (18.9%) [86]. The observed disparities may be explained by variations in study population, AMR surveillance, antimicrobial stewardship programs, and rising trends of MDR *E. coli* among human, animal, and environmental isolates [81]. The high MDR rate in all human, animal, and environmental *E. coli* isolates in our study is a significant public health challenge, highlighting the need for a molecular-based study to investigate the presence of resistance gene transmission among the human-animal-environment interface [87].

Our study also revealed that all ESBL and carbapenemase-producing isolates from patients, cattle, and the environment were MDR. This was comparable with previous studies, which reported higher MDR levels of *E. coli* in ESBL- and carbapenemase-producing *E. coli* than the non-ESBL and/or carbapenemase-producing isolates [69, 88]. Without the implementation of a multi-factorial approach under the One Health umbrella, managing patients with ESBL-PEC and CPEC MDR infection coupled with the frequent occurrence of multiple virulence factors in MDR *E. coli* [89], seems to be impossible in a country with inadequate health care infrastructure and constrained resources [90].

### Limitations

Although we documented the prevalence of ESBL-PEC in patients, cattle, and the environment for the first time based on a One Health approach, our study had some limitations. Our study did not identify the co-production of ESBL in CPEC nor the types and variants of ESBL and carbapenemases. The conventional biochemical test used for *E. coli* identification in our study exhibits a close to 10% inherent misidentification rate of bacteria to the species level compared with other identification methods, including the Matrix-assisted laser desorption ionization-time of flight mass spectrometry identification method [91, 92]. Consequently, our species-level identification may have been somewhat impacted. The sample size was determined based on the samples from a larger project. This may affect the generalizability of the finding to the larger population. Moreover, unlike the mixed time-point sample collection employed for hospital environment (before and after cleaning), all environmental samples from animal clinics were collected at a single time point (when the room was not cleaned), because there was no assigned cleaner. These variations in our sampling time may overestimate the findings in the veterinary clinic environment, since cleaning can reduce colonization of the environment with potentially pathogenic bacteria [93].

## Conclusion

The prevalence of ESBL-PEC was high among patients and the hospital environment, with a high level of MDR rates. Although the prevalence of ESBL-PEC in cattle was low, the high MDR rate of the isolates in cattle is a wake-up call to prevent further increase in the community, accustomed to eating raw foods of animal origin. However, the prevalence of CPEC was low among patients and in the hospital environment, with no CPEC isolate detected in cattle and the veterinary clinic environment. Reinforced infection prevention and control measures in hospitals, including proper wastewater management, are needed. Training on good cattle farm hygienic management practices to farm owners and farmers owning animals in the community is essential to prevent cross-transmission and further spread of MDR ESBL-PEC. The cephamycin cefotetan may serve as a potential carbapenem-sparing option to manage ESBL-PEC infections. The presence of substantial MDR *E. coli* in patients, the environment, and cattle underscores the need for implementing AMR surveillance based on the One Health approach to prevent the emergence of *E. coli* strains resulting in human and animal infections untreatable using available antibiotics. Further molecular-based studies are needed to identify the MDR *E. coli* pathotypes harbored by cattle and determine ESBL and carbapenemase types and variants in *E. coli* colonizing the human-animal-environment interface.

## Supplementary Information

Supplementary Material 1.

## Data Availability

The dataset generated during and/or analyzed during the current study is included within the manuscript and supporting information.

## Abbreviations

AMR: Antimicrobial resistance
ATCC: American Type Culture Collection
BAP: Blood agar plate
CFU: Colony forming unit
CLSI: Clinical Laboratory Standard Institute
CPEC: Carbapenemase-producing E. coli
ESBL-PEC: ESBL-producing E. coli
ExPEC: Extraintestinal pathogenic E. coli
FHCSH: Felege-Hiwot Comprehensive Specialized Hospital
InPEC: Intestinal pathogenic E. coli
IRERC: Institutional Research Ethics Review Committee
MAP: MacConkey agar plate
mCIM: Modified carbapenem inactivation method
MDR: Multidrug resistant
MHA: Muller-Hinton agar
RAMS: Rectoanal mucosal swab
TGSH: Tibebe-Ghion Specialized Hospital
TSB: Tryptone soya broth
UTI: Urinary tract infection
WHO: World Health Organization
