# Supplementary material for: Prevalence of extended-spectrum β-lactamase and carbapenemase-producing Escherichia coli from patients, cattle, and environmental sources in northwest Amhara, Ethiopia: a one health approach
Source: BMC Microbiol. 2026 Apr 22;26:586. doi: 10.1186/s12866-026-05070-z (PMC13330416; doi:10.1186/s12866-026-05070-z)
Supplement: Supplementary file 1 — Supplementary Material 1. [file 12866_2026_5070_MOESM1_ESM.docx]

**Annex-I: Hospital environment sample collection checklist**

| **Sample collection site:** Hospital environment | **Checklist filled by**: __________________________ |
| --- | --- |
| **Identification number**:______________________ | **Sample collected by**: ________________________ |
| **Collection date** (Year/month/day):_____________ |  |

| **Part-I: Checklist to collect data from different hospital environments** | | | |
| --- | --- | --- | --- |
| **S. no** | **Question** | **Response** (make a check mark “**√”**) | **Filters** |
| **101** | Hospital name for sample collection | Tibee-Ghion Specialized Hospital  Felege-Hiwot Comprensive Specialized hospital |  |
| **102** | Type of sample | Surface swab Hospital wastewater |  |
| **103** | Site of surface swab collection? | 1. Inpateint kitchen  2. Ward  3. ICU  4. OPD |  |
| **104** | Hospital wastewater collection site? | 1. Inpateint kitchen  2. Ward  3. ICU  4. Main reserviour |  |
| **105** | Do the hospital have treatment plant? | Yes  No | Go to 107 |
| **106** | What kind of wastewater treatment do the hospital implement? (>1 answer is possible) | Physical method  Chemical method  Biological method |  |
| **107** | Do the hospital have wastewater reservoirs tank? | Yes No |  |
| **108** | Is hospital wastewater treated or untreated? | Treated Untreated |  |
| **109** | Round of sample collection | 1. 1^st^ round 3. 3^rd^ round  2. 2^nd^ round 4. 4^th^ round |  |
| **110** | Time of collection | Morning (before 12 AM) Afternoon (12AM to 5PM) |  |
| **111** | Is the sampling site cleaned at the time of sample collection? | Yes No |  |

**Annex-II: Basic data collection questionnaire from “Bahir Dar Zuria Woreda animal health clinic” (English version)**

| **Sample collection site:** Animal clinic environment | **Checklist filled by**: __________________________ |
| --- | --- |
| **Identification number**:______________________ | **Sample collected by**: ________________________ |
| **Collection date** (Year/month/day):_____________ |  |

| **Part-I: Basic data collection on staff profile, animal health clinic environment and related data** | | | |
| --- | --- | --- | --- |
| **S. no** | **Questions** | **Response** | **Filters** |
| 201 | How many animal health professionals are working in the clinic in each qualification stated? | 1. DVM^⁋^: _______________  2. BSc graduates: ________  3. Diploma graduates: _____  4. Level II-IV graduates: ________  5. Certificate graduates:_________  6. Others (specify): ______________ |  |
| 202 | On average, how many individuals including animal health professionals use the room per day? (put the number for each listed from “A” to “C” | A. Cattle & small ruminant area: _______  B. Animal health professional room: ____  C. Laboratory examination room:_______ |  |
| 203 | How many animals get the service per day? | Avargae number/day: _____________  Avarage number/working day: ________  Avarage number/hollyday: ___________ |  |
| 204 | How many cattle get the service per day? | Avargae number/day: _____________  Avarage number/working day: ________  Avarage number/hollyday: ___________ |  |
| 205 | How frequent is the clinic cleaned? | ____________times per week |  |
| 206 | Time of cleaning (indicate in hours the day)? |  |  |
| 207 | Cleaning agents used? |  |  |
| 208 | How many cleaners are there? | 1.  2. |  |
| 209 | Educational level of cleaners | 1.  2. |  |
| 210 | Is there any antimicrobial resistance surveillance system? | Yes  No |  |
| 211 | How you discard expired antibiotics? | ------------------------------------------------ |  |
| 212 | How do you disinfect your needles? | Boiling Sterilization |  |
| 213 | Is the floor surface of animal treatment area cleaned (“Cattle & small ruminant area”)? | Yes  No | Go to Q216 |
| 214 | When does the floor of the animal treatment area cleaned? (“Cattle & small ruminant area”)? | Every -----------------------days |  |
| 215 | What agent will be used to clean? | Dry moping  0  Wet mopping using water only  Wet mopping using water & soap  Others (specify): -------------------------- |  |
| 216 | Where do you discard the manures? | **---------------------------------------------------** |  |

**Note**: ⁋→ Doctor of Veterinary Medicine

**Annex-III: Environmental swab sample collection tool from “Bahir Dar Zuria Woreda animal health clinic”**

| **Sample collection site**: Animal health clinic environment | **Sample collected by**: ---------------------------------- |
| --- | --- |
| **Identification number**:________________ | **Cheek list filled by**: __________________ |
| **Collection date** (year/month/day):___________ | **Sampling site**: Cleaned Not cleaned |
| **Sampling time**:  Morning (Before 12 AM)  Afternoon (Between 12 AM to 5 PM) |  |

| Sampling site |
| --- |
| 301. Cattle & small ruminant unit floor surface swab (**circle the exact site**)  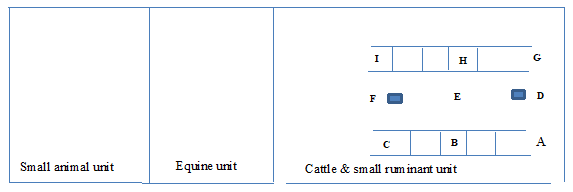 |
| 302. Animal health professional room table top surface |
| 303. Laboratory examination room table top surface |
| 304. Round of sample collection  1. 1^st^ round 3. 3^rd^ round  2. 2^nd^ round 4. 4^th^ round |

**Annex-IV: Questionnaire/checklist to collect data about the cattle included in the study**

**Preliminary: Study participant identification**

| **Study subject**:  Cattle attending animal clinic Cattle in the farm | **Questionnaire filled by**: ________________ |
| --- | --- |
| **Identification number**:________________ |  |
| **Kebele**:____________**Phone**:__________________ | **Sample type**: Rectoanal mucosal swab |
| **Sample collected by**: ---------------------------------- |  |
| **Cattle health status**  Diarrheal disease  Pneumonia  Apparently healthy | **Collection date** (Year/month/day):__________ |

| **Part-1: Questions/checklist related with cattle included in the study** | | | |
| --- | --- | --- | --- |
| **S. no** | **Questions** | **Response/entry** | **Filters** |
| **401** | Age of cattle included in the study | ------------------------(years & onths) |  |
| **402** | Breed of the cattle | Crossbreed Local  Forign purebread |  |
| **403** | Sex of the cattle | Male  Female | Go to Q407 |
| **404** | How many births do the cow give? | ----------------------(in numbers) |  |
| **405** | Does cow give multiple calves in single birth? | Yes  No | Go to Q408 |
| **406** | In how many of its birth does it give multiple calves? | ----------------------- (numbers) |  |
| **407** | Is it an ox or a bull? | Ox Bull |  |
| **408** | Is the cattle given antibiotics in the past three months? | Yes  No | The end |
| **409** | What is the name of antibiotics given? | -------------------------------------- |  |

**Annex-V**: Questionnaire to collect data from cattle/farm owners about the cattle included in the study

**Preliminary: Study participant identification**

| **Study subject**:  Cattle attending animal clinic Cattle in the farm | | **Questionnaire filled by**: __________________ | | |
| --- | --- | --- | --- | --- |
| **Identification number**:________________ | | **Sample type**: Rectoanal mucosal swab | | |
| **Kebele**:____________**Phone**:__________________ | |  |  |  |
| **Sample collected by**: ---------------------------------- | | **Collection date** (Year/month/day):___________ | | |
| **Cattle health status**  Diarrheal disease Apparently healthy  Pneumonia | | | | |
| **Part-I: questions related with cattle included in the study** | | | | |
| **S. no** | **Questions** | | **Response** | **Filters** |
| **501** | Cattle age (in years) | | ----------------------years |  |
| **502** | Cattle bread | | Local Cross bread Pure |  |
| **503** | Sex | | Female  Male | To Q514 |
| **504** | Milk yield in litters per day & milking frequency per day | | --------litters (1 times 2 times ) |  |
| **505** | How many births do the cow give? (for cows only) | | ----------------------calve/s (in numbers) |  |
| **506** | Does cow give multiple calves in single birth? | | Yes No |  |
| **507** | In how many of its birth does it give multiple calves? | | ----------------------- (numbers) |  |
| **508** | Does the cow have teat injury | | Yes No |  |
| **509** | Number of milker (in number) | | --------------------------------- |  |
| **510** | What is your milking container | | Plastic Metal Pot Kalabash |  |
| **511** | How frequent do you wash milking container | | Evere --------------days |  |
| **512** | Do you wash udder every day before milking | | Yes No |  |
| **513** | Do you dry udder after washing | | Yes No |  |
| **514** | Is it ox or bull? | | Ox Bull |  |
| **515** | What is bedding material for your cattle | | No Wood Sand Concrit |  |
| **516** | How frequent do you clean animal house (in days) | | Once in--------------day/s |  |
| **517** | Do you use free grazing feeding practice | | Yes No |  |
| **518** | For which animal do you use free grazing | | Crossbreed Local Pure |  |
